# The Chronic Migraine Brain: What Have We Learned From Neuroimaging?

**DOI:** 10.3389/fneur.2019.01356

**Published:** 2020-01-09

**Authors:** Massimo Filippi, Roberta Messina

**Affiliations:** ^1^Neuroimaging Research Unit, Division of Neuroscience, Institute of Experimental Neurology, IRCCS San Raffaele Scientific Institute, Milan, Italy; ^2^Neurology Unit, IRCCS San Raffaele Scientific Institute, Milan, Italy; ^3^Vita-Salute San Raffaele University, Milan, Italy

**Keywords:** chronic migraine, neuroimaging, chronification, pain network, biomarkers

## Abstract

Chronic migraine is a highly disabling disease with a great impact on socioeconomic functioning and quality of life of migraine patients. Chronic migraine usually evolves from episodic migraine that gradually increases in attack frequency, supporting the view of migraine as a spectrum disorder. Pathophysiological mechanisms responsible for migraine chronification are not fully understood. Likewise episodic migraine, chronic migraine patients show widespread functional and structural alterations of cortical and subcortical pain-related brain areas. However, chronic migraine patients experience a more pronounced dysfunction of the pain inhibitory network and an increased sensitization of the central pain pathways, which might explain the higher susceptibility to migraine attacks. Imaging studies have highlighted that brain regions with a key role in migraine attack generation, like the pons and hypothalamus, might also be involved in migraine chronification. Whether brain alterations are biomarkers that predispose migraine patients to chronification or reflect adaptive or maladaptive responses to the increasing headache frequency is still a matter of debate. The central mechanisms of action of chronic migraine preventive treatments and imaging biomarkers that could predict patients' treatment response have also been explored. In this new era of migraine treatments, a better understanding of chronic migraine pathophysiology will pave the way for the development of new improved treatments specifically designed for chronic migraine patients.

## Introduction

Chronic migraine is a highly disabling disease. Relative to episodic migraine, patients with chronic migraine have greater headache-related impact on socioeconomic functioning and worse quality of life (1). According to the International Classification of Headache Disorders (2), chronic migraine is defined as at least 15 days of headache occurring each month, including at least 8 days a month of headache attacks with migrainous features, for more than 3 months. The prevalence of chronic migraine is around 1–2% in the general population. Chronic migraine usually evolves from episodic migraine that gradually increases in attack frequency, with an annual progression rate of about 3% (3, 4). The main risk factors for transition are female sex, low educational status, baseline high attack frequency, obesity, stressful life events, snoring, ineffective acute treatments, and overuse of acute migraine medications (5, 6). At least 50% of patients with chronic migraine regularly overuse one or more drugs usually taken for acute migraine treatment, thus fulfilling the diagnosis of chronic migraine with medication overuse (2, 7, 8). Compared to episodic migraine patients, patients with chronic migraine are more likely to have psychiatric comorbidities, like depression and anxiety, respiratory and cardiovascular diseases (1). Chronic migraine is a dynamic state, with patients moving in and out of the chronic condition. About 26% of patients with chronic migraine remit to the episodic form within 2 years (9).

Pathophysiological mechanisms responsible for migraine chronification are not fully understood. Patients with chronic migraine might have a lower sensory threshold and an increased susceptibility to migraine attacks (3). Central and peripheral sensitization processes can contribute to the pathophysiology of chronic migraine. Of note, compared to episodic migraine patients, patients with chronic migraine have higher plasma levels of vasoactive neuropeptides, such as the calcitonin gene related peptide and vasoactive intestinal peptide, thus suggesting an altered activity of the trigeminal and cranial autonomic system (3, 10, 11). Dysfunction of cortical and subcortical brain areas involved in pain processing, such as the thalamus, hypothalamus, somatosensory and anterior cingulate cortex, might also have a pivotal role in migraine transformation. There is evidence that an altered balance between the facilitatory and inhibitory activity of pain-related brain regions might contribute to the development of symptoms commonly reported by chronic migraine patients, like cutaneous allodynia (12, 13). Our understanding of the pathophysiology of chronic migraine has improved considerably with a series of imaging studies, which have provided insights into the function and structure of human brain networks that could be involved in migraine chronification. This review will focus the attention on neuroimaging studies in patients with chronic migraine, highlighting the evidence behind the involvement of key brain areas, such as the pons and hypothalamus, and the pain network in migraine chronification. Table 1 summarizes the main findings of neuroimaging studies in chronic migraine patients.

**Table 1:** A summary of the main findings of neuroimaging studies in chronic migraine patients.

**Study**	**Study cohorts**	**Main findings**	**Potential confounders**
**RESTING STATE fMRI STUDIES**			
Androulakis et al. (14, 15)	13 CM patients without MOH (all females), 16 CM patients with MOH (all females) vs. 19 controls (all females)	Compared to controls, CM patients, regardless of MOH status, showed: - ↓ overall network connectivity of the DMN, SN and ECN Frequency of headache attacks was negatively correlated with the strength of the SN and ECN intrinsic connectivity Severity of cutaneous allodynia was positively correlated with the strength of the SN intrinsic connectivity	15 patients were taking migraine prophylaxis
Chen et al. (16)	16 CM patients without MOH, 18 EM patients vs. 21 controls	Compared to controls and EM patients, CM patients had: - ↑ RS FC between the anterior hypothalamus and the right orbital gyrus	14 CM patients had headache without migraine features during MRI scanning
Coppola et al. (17)	20 CM patients without MOH vs. 20 controls	Compared to controls, CM patients showed: - ↓ RS FC between the DMN and ECN - ↑ RS FC between the DAS and DMN - ↓ RS FC between the DAS and ECN. The severity of headache was positively correlated with the strength of the DAS intrinsic connectivity The severity of headache was negatively correlated with the strength of the ECN intrinsic connectivity	
Lee et al. (18)	19 CM patients without MOH vs. 45 EM patients	Compared to EM patients, CM patients showed: - ↑ RS FC of pain processing areas, including the anterior cingulate cortex - ↓ RS FC between pain processing brain areas and the hypothalamus - ↑ RS FC between pain processing brain areas and the dorsal raphe nucleus	
Lerebours et al. (19)	25 CM patients with MOH vs. 22 EM patients	Compared to EM patients, CM patients had: - ↑ RS FC between the anterior hypothalamus and the spinal trigeminal nucleus	16 CM patients had mild headache during the MRI 6 CM patients were taking migraine prophylaxis
Schwedt et al. (20)	20 CM patients without MOH vs. 20 controls	Atypical RS FC between: - Left anterior insula and left pulvinar, parieto-temporal areas, right precuneus, cingulate cortex and bilateral thalamus - Right anterior insula and left pulvinar, right periaqueductal gray, middle temporal cortex, and bilateral thalamus - Left amygdala and right superior frontal gyrus - Right amygdala and occipital cortex Disease duration was positively correlated with: - RS FC between bilateral anterior insula and right thalamus - RS FC between right anterior insula and right periaqueductal gray Anxiety scores were negatively correlated with: - RS FC between right anterior insula and right periaqueductal gray	8 patients were taking migraine prophylaxis
**fMRI STUDIES OF NOCICEPTIVE STIMULATION**			
Ferraro et al. (21)	9 CM patients with MOH (all females) vs. 9 controls (all females)	Compared to controls, CM patients with MOH showed: - ↓ pain-related activity of bilateral inferior parietal lobule, somatosensory cortex and right supramarginal gyrus In CM patients, the activity of pain processing regions normalized at 6 months after withdrawal	During the MRI exam, all patients had a moderate headache
Schulte et al. (22)	17 CM patients without MOH, 18 EM patients vs. 19 controls	Compared to controls, CM patients showed: - ↑ activation of the anterior right hypothalamus Compared to controls and migraineurs (EM and CM) without headache, migraineurs with headache showed: - ↑ activation of the posterior hypothalamus bilaterally	4 CM patients were taking migraine prophylaxis 19 patients (7 EM and 12 CM patients) had headache during the MRI
**fMRI STUDIES DURING A DECISION-MAKING TASK**			
Ferraro et al. (23)	8 CM patients with MOH (all females), 8 detoxified CM patients with MOH (all females), 8 CM patients without MOH (all females) vs. 8 controls (all females)	Compared to controls, CM patients with MOH showed: - ↓ task-related activity in the substantia nigra/ventral tegmental area complex - ↑ task-related activity in the ventromedial prefrontal cortex Compared to CM without MOH, CM patients with MOH showed: - ↓ task-related activity in the substantia nigra/ventral tegmental area complex Compared to detoxified MOH patients, CM patients with MOH showed: - ↑ task-related activity in the ventromedial prefrontal cortex	During the MRI exam, all patients had a moderate headache
**fMRI STUDIES OF VISUAL STIMULATION**			
Schulte et al. (24)	17 CM patients without MOH, 18 EM patients vs. 19 controls	Compared to controls, CM patients showed: - ↑ activation of the spinal trigeminal nucleus and superior colliculi	4 CM patients were taking migraine prophylaxis 19 patients (7 EM and 12 CM patients) had headache during the MRI
**POSITRON EMISSION TOMOGRAPHY STUDIES**			
Aurora et al. (25)	10 CM patients with or without MOH	CM patients had: - ↑ metabolism in the pons and right temporal cortex compared to the global cerebral metabolism - ↓ metabolism in the bilateral caudate nuclei, frontal and parietal cortex compared to the global cerebral metabolism	
Fumal et al. (26)	16 CM patients with MOH vs. 68 controls	Before withdrawal, compared to controls, CM patients with MOH showed: - ↓ metabolism of the bilateral thalamus, orbitofrontal cortex, anterior cingulate gyrus, insula, ventral striatum, and right inferior parietal lobule - ↑ cerebellar metabolism In CM patients with MOH, all dysmetabolic areas recovered to almost normal glucose uptake 3 weeks after withdrawal, except the orbitofrontal cortex where a further metabolic decrease was found	
Matharu et al. (27)	8 CM patients	No significant differences in the activity of the dorsal rostral pons in CM patients during pain and in pain-free patients during bilateral suboccipital stimulation ↓ activation of the anterior cingulate cortex in pain-free CM patients during bilateral suboccipital stimulation ↑ activation of the anterior cingulate cortex and cuneus in CM patients during pain	
**PROTON MAGNETIC RESONANCE SPECTROSCOPY STUDIES**			
Niddam et al. (28)	25 CM patients without MOH, 24 EM patients vs. 25 controls	Compared to controls, CM patients had: - ↓ N-acetyl-aspartate concentration of the right thalamus and anterior cingulate cortex - Altered interregional N-acetyl-aspartate correlations between the thalamus and anterior cingulate cortex and between the thalamus and occipital cortex in the right hemisphere Compared to controls and EM patients, CM patients had: - ↓ N-acetyl-aspartate concentration of the left thalamus In CM patients, the right thalamic N-acetyl-aspartate concentrations was negatively correlated with patients' disease duration In CM patients, there was a positive correlation between the N-acetyl-aspartate concentration and gray matter volume of the right anterior cingulate cortex	21 patients (3 EM and 18 CM patients) had headache the day of the MRI
**MORPHOMETRIC STUDIES**			
Bilgic et al. (29)	17 CM patients without MOH (all females), 7 CM patients with MOH (all females) vs. 24 controls (all females)	Compared to controls, CM had: ↓ cerebellar and brainstem volume	7 patients were taking migraine prophylaxis
Chen et al. (16)	16 CM patients without MOH, 18 EM patients vs. 21 controls	Compared to controls and EM patients, patients with CM showed: - ↓ volume of the anterior hypothalamus In CM patients, the anterior hypothalamic volume was positively correlated with headache frequency Cut-off volume of the hypothalamus as 1.429 ml had a good diagnostic accuracy for CM with sensitivity of 81% and specificity of 100%	14 CM patients had headache without migraine features during MRI scanning
Coppola et al. (30)	20 CM patients without MOH vs. 20 controls	Compared to controls, CM patients had: - ↓ gray matter volume of the right cerebellum, left pallidum, amygdala, orbitofrontal, temporal, and occipital cortex In CM patients, the cerebellar gray matter volume was: - Negatively correlated with patients' disease duration - Positively correlated with the number of acute medications taken per month	4 patients had mild headache without migrainous features during the MRI exam
Hubbard et al. (31)	23 CM patients (11 responders and 12 non-responders to prophylactic treatment with onabotulinumtoxinA)	Compared to non-responders, patients who responded to onabotulinumtoxinA showed: - ↑ cortical thickness of the right primary somatosensory cortex, anterior insula, left superior temporal gyrus and pars opercularis In responders patients, disease duration was: - Negatively correlated with cortical thickness of fronto-parietal and temporo-occipital regions - Positively associated to the cortical thickness of the left primary motor cortex In non-responders patients, disease duration was: - Negatively associated to the cortical thickness of the left primary motor cortex - Positively associated to the cortical thickness of the left inferior temporal gyrus and lateral occipital cortex	Some patients in the non-responder group may have had mild headache the day of the MRI
Lai et al. (32)	33 CM patients with MOH (19 responders to common preventive treatments), 33 CM patients without MOH vs. 33 controls	Compared to CM patients without MOH, patients with MOH showed: - ↓ gray matter volume of the bilateral orbitofrontal cortex and left middle occipital gyrus - ↑ gray matter volume of the left temporal pole/parahippocampus In CM patients with MOH, clinical improvement after 12 months of preventive treatment was significantly associated to the gray matter volume of the orbitofrontal cortex In CM patients, gray matter volume changes could predict the frequency of analgesics use	33 patients had migraine the day of the MRI exam (13 CM patients without MOH and 20 CM patient with MOH)
Liu et al. (33)	39 CM patients, 83 EM patients (15 patients with MOH) vs. 31 controls	In CM and EM patients, the volume of the bilateral hippocampus and left amygdala varied as a function of headache frequency At 2-year follow-up, the volume of the right hippocampus was positively associated with a good migraine outcome	
Neeb et al. (34)	6 CM patients without MOH, 15 CM patients with MOH, 21 EM patients vs. 21 controls	Compared to controls, CM patients had: - ↑ gray matter volume of the right amygdala, superior parietal lobule, hippocampus, parahippocampus, left insula, and bilateral basal ganglia Compared to EM patients, CM patients showed: - ↑ gray matter volume of bilateral temporal areas - ↓ gray matter volume of the left cuneus In CM and EM patients, gray matter volume alterations were influenced by headache frequency	13 patients (9 CM and 4 EM patients) were taking migraine prophylaxis
Niddam et al. (28)	25 CM patients without MOH, 24 EM patients vs. 25 controls	Compared to controls, CM patients had: - ↓ gray matter volume of the right anterior cingulate cortex	
Riederer et al. (35)	31 CM patients with MOH (10 responders and 8 non-responders to medication withdrawal)	At baseline, compared to responder patients, non-responders had: - ↓ gray matter volume of the right orbitofrontal cortex Only responders patients showed ↓ gray matter volume of the midbrain after medication withdrawal Treatment response correlated positively with: - Baseline gray matter volume of the orbitofrontal cortex - Gray matter volume change in the midbrain after medication withdrawal	20 patients were taking migraine prophylaxis
Schwedt et al. (36)	15 CM patients without MOH, 51 EM patients vs. 54 controls	Average accuracy of classifiers consisting of cortical surface area, cortical thickness, and regional volumes of fronto-temporal areas was: -86.3% for CM patients vs. controls - 84.2% for CM vs. EM patients - 67.2% for EM patients vs. controls	

*CM, chronic migraine; DAS, dorsal attention system; DMN, default mode network; EM, episodic migraine; ECN, executive control network; fMRI, functional magnetic resonance imaging; MOH, medication overuse headache; RS FC, resting state functional connectivity; SN, salience network*.

## Imaging the Pain Network in Chronic Migraine

Pain experience is a complex process involving sensory, affective, and cognitive brain networks. Similar to previous findings in episodic migraine patients (37), an altered functional recruitment of brain areas involved in the sensory-discriminative and affective aspects of pain, including the insula, prefrontal, anterior cingulate and somatosensory cortex, has been demonstrated in chronic migraine patients (20). Maladaptive functional activation of brain networks involved in attentive and executive functions, such as the executive control, default mode and dorsal attention network, have also been revealed in patients with chronic migraine. Thus, suggesting that the reaction to painful stimuli, preparation of responses, and allocation of attentional resources to pain are impaired in chronic migraine patients (14, 15, 17). Whether cognitive symptoms, particularly deficits in attention and executive functions, might influence the functional activity of brain cognitive networks has never been investigated. A comprehensive neuropsychological assessment should be included in future studies. The salience network has a key role in defining the saliency of incoming painful stimuli. In chronic migraine patients, the presence of cutaneous allodynia was associated to an increased activity of the salience network. These findings support a possible involvement of the salience network in central sensitization (14).

Several studies (29, 30, 34) demonstrated that chronic migraine is also associated with morphometric alterations of brain areas known to be involved in pain modulation and in the different aspects of pain processing. Regions of increased and decreased gray matter volume, including the brainstem, cerebellum, basal ganglia, amygdala, frontal, temporal and occipital areas, have been found in chronic migraine patients compared to controls (29, 30, 34).

Whether these functional and structural alterations are the consequence of the recurrence of headache attacks or might predispose to chronic migraine is still a matter of debate. Some studies demonstrated functional (14, 20) and structural (30, 33, 34) plasticity of nociceptive brain areas that are linked to the headache attack frequency and disease duration. Repetitive headache attacks can remodel the pain network, thus increasing the susceptibility to the onset of further attacks and leading to chronic central sensitization. On the other hand, other investigations did not confirm such correlation (29).

Dynamic functional (21, 26) and structural (32, 35) changes in pain processing structures were also revealed in chronic migraine patients with medication overuse. Interestingly, imaging alterations of the thalamus, insula, anterior cingulate, and parietal cortex reverted after medication withdrawal, probably reflecting the consequences rather than the causes of medication overuse in these patients. While alterations of mesocorticolimbic dopaminergic areas, such as the ventral tegmental area (23) and orbitofrontal cortex (26), persisted following detoxification, suggesting that these findings might represent a brain trait that predisposes certain migraine patients to the development of medication overuse.

Quantitative MRI techniques have shown increased iron deposition in the periaqueductal gray, red nucleus, and basal ganglia in migraine patients and patients with chronic daily headache (38–40). A higher risk to have iron deposition was associated to higher attack frequency or longer disease duration, suggesting a causal relationship between migraine and these abnormalities (38). The observed association between repeated migraine attacks and increased iron accumulation in the brainstem and deep gray matter nuclei involved in central pain processing support the possibility that migraine has cumulative effects on brain structure and homeostasis. However, a follow-up study did not find any significant progression of iron accumulation over 9 years (41).

A further unanswered question is whether neuroimaging alterations are common to episodic and chronic migraine patients or are specifically involved in migraine chronification. There is evidence showing a more pronounced dysfunction of the pain inhibitory (18) and thalamocortical (28) pathway in chronic than episodic migraine. Using resting state functional MRI, Lee and coworkers (18) have shown an increased functional connectivity of pain processing brain areas, especially the anterior cingulate cortex, in chronic migraine patients compared to patients with episodic migraine (18). Reduced N-acetyl-aspartate concentration in the thalamus and anterior cingulate cortex has been found in chronic migraine patients, but not in patients with episodic migraine (28). Interestingly, the interregional correlations of N-acetyl-aspartate levels between the thalamus and the anterior cingulate cortex shifted from positive in controls to negative in chronic migraine patients. Thus, suggesting that neuronal reorganization in the thalamocortical pathway might contribute to migraine chronification.

Chronic migraine is also associated to more extensive brain structural alterations. Schwedt and colleagues (36) reported that alterations of cortical thickness, cortical surface area and regional volumes of fronto-temporal brain areas could discriminate chronic migraine patients from controls and from patients with episodic migraine with an accuracy of 86 and 84%, respectively. While, the accuracy for discriminating episodic migraine patients from controls was of only 67%. A greater iron accumulation was found in chronic migraine patients compared to patients with episodic migraine (39, 40). Larger volume of iron deposits could identify chronic migraine with a sensitivity ranging from 80 to 93% and a specificity ranging from 71 to 97% (40). The increased iron levels in the anti-nociceptive network in chronic migraine patients might constitute a physiologic response to repeated activation of nuclei involved in central pain processing, which may play a role in the chronification of migraine.

In migraine patients, the perception of the headache pain can be exacerbated by the exposure of lights. There is evidence showing that photic signals coming from the retina can converge on thalamic trigeminovascular neurons that project to cortical areas involved in the processing of pain and visual perception. Thus, supporting the link between the visual and trigeminal pain processing system (42). Interestingly, compared to controls and episodic migraine patients, chronic migraine patients showed and increased activity of the spinal trigeminal nucleus and superior colliculi during visual stimulation with a rotating checkerboard. The increased trigeminal activation during visual stimulation was significantly influenced by the experience of headache. These findings corroborate the crosslink between the visual and trigeminal systems and demonstrate a more pronounced sensitization of these two pathways in patients with chronic migraine (24).

## Imaging the Migraine “Generators” in Chronic Migraine

Although our understanding of the pathophysiology of migraine has progressed over the last years, where exactly migraine attacks originate is still an unresolved question. Several studies demonstrated a selective activation of the dorsal pons during spontaneous (43, 44) and nitroglycerin-triggered (45) migraine attacks, which persisted after complete pain-resolution due to triptan administration (44), in patients with episodic migraine. Thus, leading the authors to hypothesize that this brainstem region might represent the so-called migraine “generator.” An increased cerebral metabolism in the pons has also been described in patients with chronic migraine during and outside the headache phase (25, 27). Similar to episodic migraine, the dysfunctional activation of this brainstem region did not change after electrical suboccipital stimulation, supporting the key role of this region in migraine attack generation as well as in migraine chronification (27).

Recent MRI studies have pointed the attention to the role of the hypothalamus in migraine attack generation. Positron emission tomography (46) and functional MRI (43) studies revealed increased hypothalamic activity before and during the headache phase of the migraine attack in episodic migraine patients. An altered functional coupling between the hypothalamus and the spinal trigeminal nucleus during the precital phase and between the hypothalamus and the pons during the ictal phase have also been demonstrated (43). These findings suggest that the hypothalamus-brainstem network might be the real driver of migraine attacks.

Different regions of the hypothalamus seem be involved in the onset of the migraine attack and in migraine chronification. Chronic migraine patients with and without medication overuse showed a selective increased activity of the anterior hypothalamus during trigeminal painful stimulation (22) and in a rest condition (16, 19), compared to controls and patients with episodic migraine. Thus, suggesting that the anterior hypothalamus plays a crucial role in the pathophysiology of chronic migraine. While, the most posterior hypothalamic part was specifically linked to the acute headache phase of the migraine attack (22).

Relative to episodic migraine, an increased activation of the hypothalamus seems to facilitate the recruitment of cortical areas involved in pain processing in chronic migraine patients (18).

In conjunction with functional alterations, structural plasticity of the anterior hypothalamus has been demonstrated in patients with chronic migraine. A hypothalamic volume lower than 1.43 ml had a good diagnostic accuracy for chronic migraine with sensitivity of 81% and specificity of 100% (16).

## Imaging Biomarkers of Treatment Response in Chronic Migraine

Imaging techniques can provide new insights into the central mechanisms of action of treatments commonly used for chronic migraine. A positron emission tomography study (27) showed significant functional modulation of brain regions involved in the affective aspects of pain, including the anterior cingulate cortex and cuneus, during bilateral electrical suboccipital stimulation in a small cohort of chronic migraine patients.

Now that new mechanism-based treatments specific for migraine are available a better prediction of treatment response might facilitate the selection of the most appropriate treatment for each patient. Chronic migraine patients who responded to OnabotulinumtoxinA treatment, as evidenced by reversal from a chronic to an episodic state, had distinct patterns of morphometric and functional alterations in pain processing areas compared to those patients who did not respond (31). The identification of the brain pathways that are involved in disease reversal or progression can lead to a better understanding of the mechanisms underlying the migraine chronification. All of this is critical to discovering new treatments that prevent or slow the progression to chronic migraine. Imaging biomarkers that could predict patients' treatment response have also been identified in chronic migraine patients with medication overuse. Rieder and coworkers (35) showed significant volumetric changes in the midbrain after the withdrawal of acute headache medications only in those patients who responded to the treatment. Moreover, decreased gray matter volume of the orbitofrontal cortex predicted a negative response to detoxification.

## Conclusions

Significant advances in our understanding of chronic migraine pathophysiology have been made over the last years. Neuroimaging findings support the view that migraine is a spectrum disorder, with clinical and pathophysiological features that can progress over time. Chronic and episodic migraine share similar functional and structural alterations in brain regions implicated in the generation of the migraine attack and in pain processing. However, chronic migraine patients experience a more pronounced dysfunction of the pain inhibitory network and an increased sensitization of the central pain pathways, which might explain the higher susceptibility to migraine attacks. Whether brain alterations are biomarkers that predispose migraine patients to chronification or reflect adaptive or maladaptive responses to the increasing headache frequency is still debated. Longitudinal studies including large sample size of patients with episodic and chronic migraine are warranted. Future studies combining multimodal data, such as functional MRI, structural MRI and electroencephalographic data, might help us to achieve a better understanding of chronic migraine pathophysiology. In the future, imaging patterns that predict whether an episodic migraine patient will evolve to a chronic form should be identified. This might lead to an early prevention of migraine transformation. In this new era of migraine treatments, a better understanding of chronic migraine pathophysiology will pave the way for the development of new improved treatments specifically designed for chronic migraine patients.

## Author Contributions

MF and RM contributed to the study concept and drafting/revising the manuscript.

### Conflict of Interest

The authors declare that the research was conducted in the absence of any commercial or financial relationships that could be construed as a potential conflict of interest.
